# Spatiotemporal characteristics of an attacker’s strategy to pass a defender effectively in a computer-based one-on-one task

**DOI:** 10.1038/s41598-019-54012-5

**Published:** 2019-11-21

**Authors:** Kazushi Tsutsui, Masahiro Shinya, Kazutoshi Kudo

**Affiliations:** 1 Department of Life Sciences, Graduate School of Arts and Sciences, The University of Tokyo, Tokyo, Japan; 2 Graduate School of Integrated Arts and Sciences, Hiroshima University, Hiroshima, Japan; 3 Graduate School of Interdisciplinary Information Studies, The University of Tokyo, Tokyo, Japan

**Keywords:** Decision, Intelligence, Perception, Human behaviour, Animal behaviour

## Abstract

For modern humans, chase-and-escape behaviors are fundamental skills in many sports. A critical factor related to the success or failure of chase-and-escape is the visuomotor delay. Recent studies on sensorimotor decision making have shown that humans can incorporate their own visuomotor delay into their decisions. However, the relationship between the decision of an attacker and the visuomotor delay of a defender is still unknown. Here, we conducted a one-on-one chase-and-escape task for humans and investigated the characteristics of the direction changes of the attacker and the responses of the defender. Our results showed that the direction change of the attacker has two characteristics: uniformity of spatial distribution and bimodality of temporal distribution. In addition, we showed that the response of the defender did not depend on the position but it was delayed to the direction change of the attacker with a short interval. These results suggest that the characteristics of direction change of an attacker increased unpredictability, and it could be useful for preventing the predictive response of the defender and to receive the benefit of an extra response delay of tens of milliseconds, respectively.

## Introduction

Our decisions are often made in competitive interactions with others. In such situations, agents must achieve their own purpose in the interaction despite the conflicting purposes of other agents. A typical example of a competitive interaction is chase-and-escape behavior. Chase-and-escape interactions are ubiquitous in nature (predator–prey), and similarly feature in many sports such as football, rugby, and basketball (defender–attacker)^1–3^. In these interactions, the decisions of the agents on when and where to move are essential for survival and success^4,5^. Thus, it is important to consider how the agents make such decisions in these competitive interactions.

Decisions regarding direction changes are important in chase-and-escape interactions. Previous studies on many animals, ranging from dragonflies^6–8^, to fish^9^ and dogs^10^, to humans^11–13^, have shown that a strategy called constant bearing is commonly employed to intercept a moving target, such as prey, a frisbee, or a flying ball. When the speed of the pursuer is equal to or faster than that of the evader, this interception strategy is theoretically unbeatable without a sensorimotor delay^14^. This time delay, which is the latency from the sensory input to the motor output, is inevitable in animals and can be several hundred milliseconds in humans^15^. Consequently, from the perspective of the evader, lengthening (or at least not shortening) the visuomotor delay (response time) of the pursuer will lead to a successful escape^16^.

One possible evader strategy is to increase the unpredictability of its actions. In several studies on interceptive behaviors in severe time constraints such as in cricket, tennis, and handball, it has been reported that a pursuer (receiver or goalkeeper) anticipates the movement of the opponent using prior knowledge (experience), which is a probability distribution accumulated by long periods of observation^17–19^. For example, in a handball penalty shot, when there is a probability bias in the shot direction of the opponent, the goalkeeper is more likely to respond in that direction than to respond as if the shot of the opponent is equally likely to occur in either direction^19^. These studies suggested that the pursuer should use the situational (event) probability information to anticipate the movement of the opponent in interceptive behaviors. Given that the defender predicts the movement of the attacker to compensate for its own visuomotor delay, it should be useful for the attacker to increase the unpredictability of its own movements against the defender.

Another possible strategy of the evader is to increase efficiency. In the psychological refractory period paradigm, in which two stimuli are presented in close succession, it has been shown that the response time to the second stimulus increases if the time between stimuli is short (400 ms or less)^20–24^. From the stimulus-response viewpoint, if the two consecutive movements change the directions of the attacker, such as moving right to left to right, the response of the defender to the second direction change could be delayed. Given that the response of defender to the direction change of the attacker is delayed in such a situation, the attacker could iterate effective direction changes.

To address these possibilities, we examined probability distributions regarding the direction change of the attacker and the response time of the defender. In this study, we conducted a computer-based one-on-one task, which eliminates kinematic information to focus on the situational probability information. Our results suggest that the direction changes of the attacker have effective spatiotemporal characteristics that combine both of the above two possibilities to pass the defender.

## Methods

### Participants

Twelve participants (mean age ±SD = 24.9 ± 2.3 years) who exercised regularly participated in the experiment. All participants were right-handed, had normal or corrected-to-normal vision, and were neurologically healthy. They each received 1000 yen per hour as a reward. The study was conducted in accordance with the Declaration of Helsinki and approved by the Ethics Committee of the University of Tokyo of Arts and Sciences. Informed consent was received from each participant before the experiments. In both experiments, participants were recruited in pairs, and each member of each pair took on the roles of both the attacker (evader) and the defender (pursuer) in turn.

No statistical methods were used to predetermine sample sizes. To estimate the achieved statistical power (1 $-\beta$) a post hoc analysis was carried out. Considering the spatial uniformity of the frequency of the direction changes of the attacker as one of the primary outcomes, the achieved power was 0.26 for two-way repeated measures ANOVA. The relatively low power achieved is discussed as a limitation of the present study.

### Experimental apparatus

Participants were seated in chairs and manipulated the joystick of a controller (Xbox One) to control a disk (filled circle) on the screen. The central position of the disk was recorded on a computer (Sony SVF152C16N) running Psychtoolbox 3.0 software at a frequency of 60 frames/s and a resolution of 1366 × 768 pixels. The stimuli were presented in a lit room on a 15.5 in (34.3 × 19.3 cm) screen. The participants were seated at a viewing distance of 50 cm. A partition prevented direct viewing of the hands of the other player.

### Experimental design

Participants controlled either a red disk representing an attacker or a blue disk representing a defender on the screen. The dimensions of the onscreen court were 33.1 cm × 16.6 cm (width × height). The diameter of each disk was 1.0 cm. The start location of the attacker (red disk) was the upper middle, and that of the defender (blue disk) was the center of the court. The objective of the attacker was to move past the defender and reach the end line, which was on the lower side of the court (a yellow line) behind the defender. The objective of the defender was to catch the attacker without the attacker reaching the end line. We regarded a “catch” as a situation in which the outer edges of the disks were in contact. If the attacker left the bounds of the court, the trial was deemed a defensive success. The velocity of each agent was determined by the degree of inclination of the joystick on the respective controllers. The maximum speeds of the attacker and the defender were set equally. The experimental task began with a start cue. No additional instructions, such as a time limit, were given to the participants. To provide feedback on the result of each trial to the participants, when the attacker reached the end line (a successful attack), a high-pitched beep was played. Conversely, when the defender caught the attacker, or the attacker left the bounds of the court (a successful defense), a low-pitched beep was played. The number of successful attacks was indicated at the end of each block; blocks consisted of 30 trials.

### Experimental conditions

We used two experimental conditions in this study (slow and fast) to examine the speed dependency of agent decision making. In previous studies, it has been reported that the required movement speed (time constraint) may affect the interception strategies (reactive or predictive) of the agent^18,25^. In a one-on-one sports situation, as the movement speeds of the agent increase, it becomes more difficult for the defender to intercept the attacker^26^. Thus, to test the possibility that changes in the strategy of the defender (and changes in the strategy of the attacker corresponding to that of the defender) depended on the movement speeds of the agent, we set two speed conditions. The slow condition was set so that the defender could easily intercept the attacker, and the fast condition was set so that the possibility of interception (successful defense) or penetration (successful attack) of the two agents was balanced. The minimum speed of the agents (both attacker and defender) was 0 cm/s in both conditions, and the maximum speed of the agents was 3 cm/s in the slow condition and 4.5 cm/s in the fast condition. There were 40 warm-up trials and 240 experimental trials per pair of participants. The experimental trials were presented in 8 blocks of 30 trials each. For each condition, participants performed 4 blocks (60 trials in each role of attacker and defender; 120 trials total). The role of each participant was randomized between blocks, and the experimental condition was randomized between pairs. The experiment lasted approximately an hour.

### Data analysis

All data analysis was performed in MATLAB (MathWorks). We recorded the onscreen *X* and *Y* positions of the attacker and defender during the trial. We analyzed only the positional data collected while the absolute angle between the defender and the attacker was in the range of 0–180° to exclude situations in which the defender had given up trying to catch the attacker.

The change-direction time was defined as the time when the velocity in the *X* direction crossed zero, and the position at that time was defined as the change-direction position.

The response time was defined as the time between the change-direction time of the attacker and that of the defender. We distinguished between positive and negative X velocities. We limited the range of response times from 0 ms to 500 ms and removed any response times longer than 500 ms from the analyses to exclude responses in which the defender had given up trying to catch the attacker. The proportion of responses passed this criterion was 0.99 ± 0.01.

The frequency was defined as the average number of occurrences per second. In other words, the frequency was calculated by dividing the number of direction changes by the time over which they spent.

The standard deviation (SD) of the frequency was calculated using the frequency value in each *X* column. In this case, we divided the court into 10 *X* columns and used the frequency values in the middle 6 columns to exclude the columns containing missing values. These missing values indicated that the attacker did not move to those *X* columns.

In the analysis of the temporal aspect, the direction change was classified into two types depending on the time elapsed from the previous direction change: short-interval direction changes (<350 ms) and long-interval direction changes (>350 ms). The boundary between them was 350 ms in both conditions. To determine this boundary, we fitted a mixture model containing a multimodal Gaussian distribution. The number of Gaussian distributions was 4, which was determined using Akaike’s Information Criterion. We estimated the parameters of this model using maximum likelihood estimates.

### Statistical analysis

To compare the successful attack rates between conditions, we used the paired *t*-test because the normality assumption was accepted by Lilliefors test. To compare the frequency of the direction changes made by the attacker, a two-way repeated measures ANOVA with the factors of speed condition (slow, fast) and *X* position was used. In this comparison, the *X* position was divided into 10 columns, and we used the middle 6 columns in the statistical test to remove the columns containing missing values caused by the attacker never changing direction in those columns. To compare the SD of the frequency of direction changes of attacker, a two-way repeated measures ANOVA with the factors of speed condition and *X* position and with the factors of speed condition and number of trials was used. In this case, the term “number of trials” indicated the number of cumulative trials, and the comparison was made among 0 to 10, 0 to 20, 0 to 30, 0 to 40, 0 to 50, and 0 to 60 trials. To compare the response time of the defender, two-way repeated measures ANOVAs with the factors of speed condition and number of trials was used. In this comparison, the *X* position was divided into 10 columns, and we use the middle 4 columns in the statistical test to remove columns containing missing values caused by the defender failing to respond to the direction change of the attacker in those columns. In this case, the term “number of trials” indicated 10 trials, and the comparison was made among 0 to 10, 11 to 20, 21 to 30, 31 to 40, 41 to 50, and 51 to 60 trials. To compare the response time of the defender, a two-way repeated measures ANOVA with the factors of speed condition and the time interval (time from the previous direction change of the attacker) was used. In this case, we removed one participant from the statistical test because the participant included missing values caused by the opponent (attacker) never changing direction during the time interval. In the ANOVAs, Greenhouse-Geisser correction was applied for the violations of sphericity assumption in the Mauchly test. Multiple comparisons with Bonferroni correction were applied in the *post-hoc* analysis. To compare the distribution of positions of change-direction with the short-interval and that with the long-interval, the Kolmogorov–Smirnov test was used. The significance level was set at *p* < 0.05 and adjusted with the Bonferroni correction in the multiple comparisons. The effect size was estimated using Cohen’s *d* for *t*-test and multiple comparison and eta-squared (*η*^2^) for ANOVA. All data are reported as mean ± SD across subjects. Statistical analyses were performed using the R version 3.5.1 and G*power version 3.1^27^.

## Results

The successful attack rates were 14.2 ± 13.2% in the slow condition and 51.8 ± 11.8% in the fast condition (*t*_11_ = 7.37, *p* = 1.4 × 10^−5^, *d* = 3.02).

**Figure 1:**
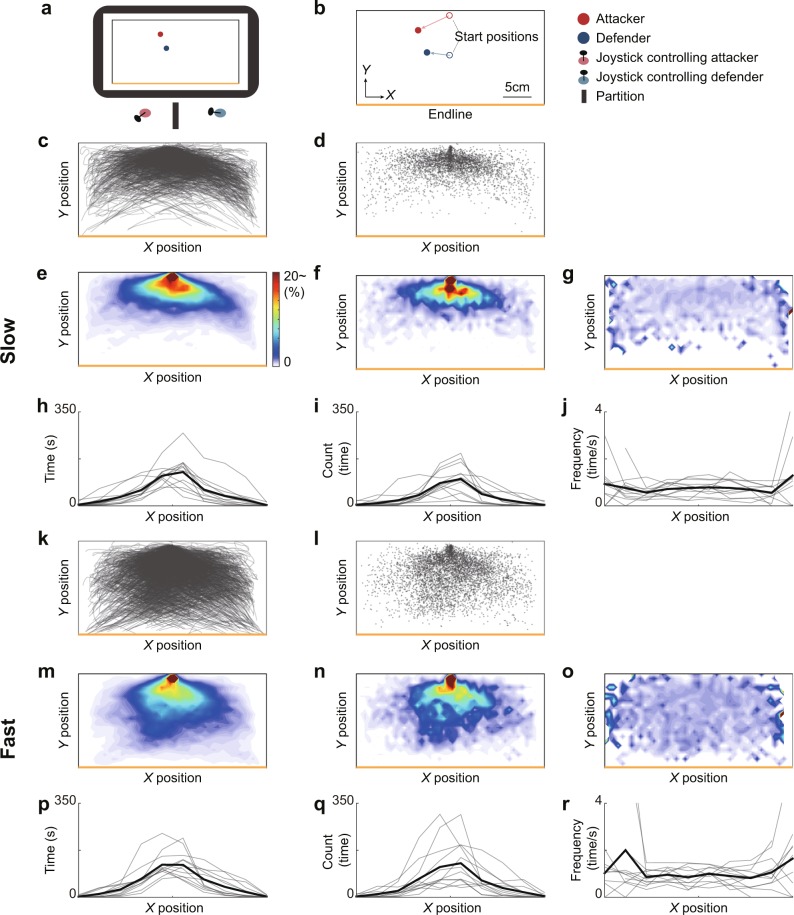
Experimental methods and spatial characteristics regarding direction changes of the attacker. (**a**) Illustration of experimental setup. Participants controlled either a red disk representing an attacker or a blue disk representing a defender on the screen using a joystick. A partition prevented direct viewing of the hands of the other participant. (**b**) Experimental task. The start location of the attacker was the upper middle (red circle) and that of the defender was the center (blue circle) of the court. The objective of the attacker was to move past the defender and reach the end line (yellow). The objective of the defender was to catch the attacker (contact the outer edges of the disks). If the attacker left the bounds of the court, the trial was deemed a defensive success. The velocity of each agent was determined by the degree of inclination of the joystick on the respective controllers. (c–j) Results of the slow condition and (k–r) those of the fast condition. (c, k) Overwriting of the trajectories of the attackers from all trials, and (d, l) 2D-histograms at each position. The court was divided into 968 (44 × 22) cells of 30 square pixels each. (e, m) Histograms of attacker time spent in different *X* positions for each participant. The *X* position was divided into 10 columns. (f, n) The direction change points in the *X* direction of the attackers from all trials, and (g, o) 2D-histograms at each position. (h, p) Histograms of the direction changes in the *X* position of each participant. (i, q) The frequency of direction changes at each position from all trials (i = g/d, q = o/l). (j, r) The frequency of direction changes in *X* position of each participant (j = h/e, r = p/m).

We illustrated the overwriting of the trajectories of the attackers from all trials in each condition (Fig. 1c,k) and the 2D-histogram (i.e., heatmap) at each position (Fig. 1e,m). In this case, the court was divided into the 968 (44 × 22) cells of 30 square pixels each. Then, we plotted the change-direction positions of the attackers (Fig. 1d,l) and the 2D-histogram at each position (Fig. 1f,n). The frequency of direction changes at each position was almost uniform (Fig. 1g,o). We focused on the data in the *X* direction and quantified them. Figure 1h,p show the time that the attacker spent within each *X* column, and Fig. 1I,q show the numbers of direction changes. Figure 1j,r show the frequency of direction changes. A two-way (condition and *X* position) repeated measures ANOVA revealed a main effect of the condition (*F*_1, 11_ = 13.29, *p* = 0.0039, *η*^2^ = 0.068). Notably, however, the main effect of the *X* position and the interaction between these factors were not significant (*F*_5, 55_ = 1.905, *p* = 0.11, *η*^2^ = 0.028; *F*_5, 55_ = 0.791, *p* = 0.56, *η*^2^ = 0.0093).

**Figure 2:**
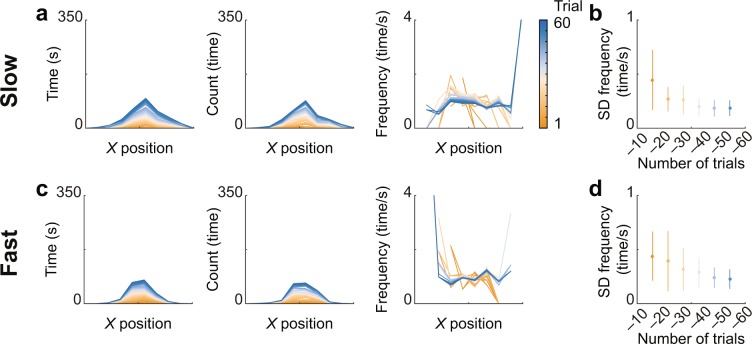
Cumulative changes in frequency regarding the direction changes of the attacker. (**a,b**) Results from the slow condition and (**c,d**) from the fast condition. (**a,c**) Representative examples of the cumulative change, and (**b,d**) the group data of the standard deviation (SD) of the frequency of direction changes in each *X* column of the cumulative change.

Figure 2a,c show typical examples of attacker data for a participant in each condition. Each figure shows the cumulative changes in the time spent in the column (left), in the number of direction changes (middle), and in the frequency (right) of the attacker in each *X* column through all trials. In the SD of the frequency between *X* columns, a two-way (condition and trials) repeated measures ANOVA revealed a main effect of trials (*F*_1.97, 21.68_ = 11.72, *p* = 4.0 × 10^−4^, *η*^2^ = 0.21). The conditions and the interaction between these factors were not significant (*F*_1, 11_ = 1.51, *p* = 0.25, *η*^2^ = 0.029, 1 $-\beta$ = 0.54; *F*_2.31, 25.37_ = 1.171, *p* = 0.34, *η*^2^ = 0.013). As the main effect of the trials was significant, we conducted *post hoc* analysis (with Bonferroni correction), and the results revealed that the SD of the frequency up to 10 trials was significantly larger than the others, except for up to 20 trials. (Fig. 2b,d; *ps* < 0.0013, *ds* > 0.74). This result indicates that the SD of the frequency decreased initially and did not change thereafter.

**Figure 3:**
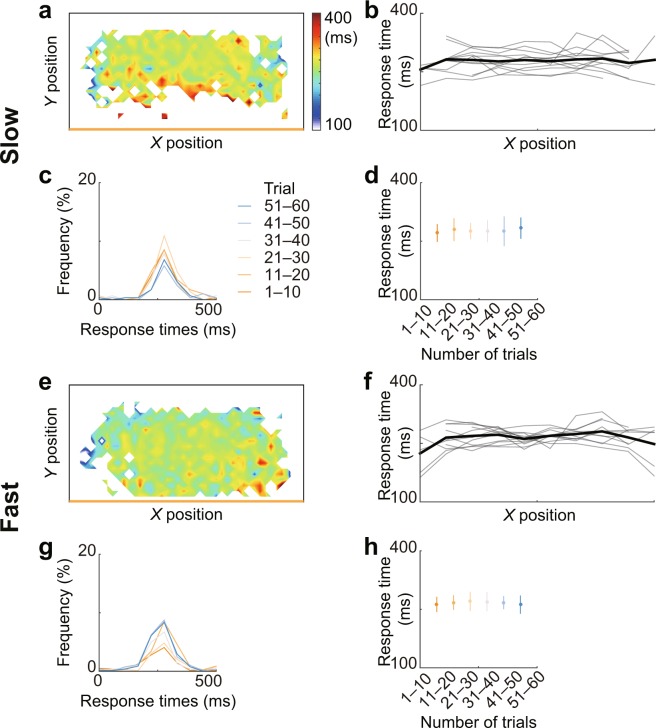
Response time of the defender to direction changes of the attacker. (**a**–**d**) Results from the slow condition and (**e**–**h**) from the fast condition. (**a,e**) Mean response times of the defender in each position from all trials. The court was divided into 968 (44 × 22) cells of 30 square pixels each. (**b,f**) The mean response time in *X* position of each participant. In this case, the *X* position was divided into 10 columns. (**c,g**) Representative example histogram of the response time over time. Each color represents a total of 10 trials. (**d,h**) The group data of the mean response time in each of the 10 trials over time.

Figure 3a,e show the mean response times of the defender to the direction changes of the attacker at each position. A two-way (condition and *X* position) repeated measures ANOVA revealed that the main effects and the interaction were not significant (Fig. 3b,f; *F*_1, 11_ = 2.37, *p* = 0.15, *η*^2^ = 0.047; *F*_1.52, 16.69_ = 0.69, *p* = 0.48, *η*^2^ = 0.013; *F*_3, 33_ = 1.68, *p* = 0.19, *η*^2^ = 0.014). Figure 3c,g show typical histogram examples of the response times of the defender in each of the 10 trials, which competed with the participant in Fig. 2a,c, respectively. A two-way (condition and trials) repeated measures ANOVA revealed that the main effects and the interaction were not significant (Fig. 3d,h; *F*_1, 11_ = 2.31, *p* = 0.16, *η*^2^ = 0.047; *F*_5, 55_ = 0.641, *p* = 0.67, *η*^2^ = 0.0094; *F*_5, 55_ = 1.141, *p* = 0.35, *η*^2^ = 0.013).

**Figure 4:**
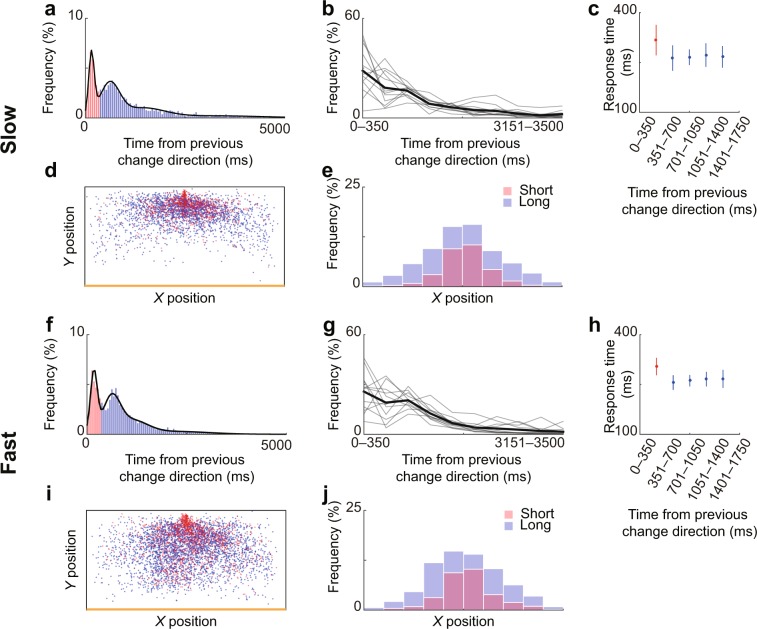
Temporal characteristics regarding direction changes of the attacker and response time of the defender. (**a**–**e**) Results from the slow condition and (**f**–**j**) from the fast condition. (**a,f**) The relative frequency (probability distribution) of time delays from the previous direction change. The direction changes were classified into short-interval (red) and long-interval (blue) at 350 ms. (**b,g**) Histograms of the time delay from the previous direction change of each participant. The time range was set from 0 to 3500 ms, and the range was divided into 10 separate time bins. (**c,h**) The group data of the mean response time in each time bin. To exclude time bins containing a small number of data, the range of the time bins was limited from 0 to 1750 ms. (**d,i**) The points of short-interval direction changes (red) and long-interval direction changes (blue) from all trials, and (**e,j**) their histograms at each *X* position.

We next examined the time interval of the direction changes of the attacker. Figure 4a,f show the relative frequency of change-direction times from the previous change-direction time. The distributions were bimodal in both conditions, and thus, we classified the directions changes into two types: short-interval and long-interval (red and blue parts in Fig. 4a,f, respectively). The boundary between them was 350 ms in both conditions (see Methods).

For the response time of the defender to the direction change of the attacker, a two-way (condition and time interval) repeated measures ANOVA revealed a main effect of time interval (*F*_4, 40_ = 17.2, *p* = 2.8 × 10^−8^, *η*^2^ = 0.32). The condition and the interaction between these factors were not significant (*F*_1, 10_ = 0.80, *p* = 0.39, *η*^2^ = 0.0059; *F*_1.62, 40_ = 0.31, *p* = 0.69, *η*^2^ = 0.0053). As the main effect of trials was significant, we conducted *post hoc* analysis (with Bonferroni correction), and the results revealed that the response time of the defender to the short-interval direction changes of the attacker was significantly greater than to the others (Fig. 4c,h; *ps* < 1.7 × 10^−6^, *ds* > 1.4). The response times to the direction change with short-interval and with long-interval were 317 ± 45 and 270 ± 20 ms in the slow condition, and those were 304 ± 25 and 264 ± 17 ms in the fast condition, respectively. The short-interval change-direction positions were distributed more in the center of the field compared with those with long intervals (Fig. d,e,i,j; *p* = 2.1 × 10^−33^).

## Discussion

Here, we explore how agents make their decisions in complex interactions such as those seen in a variety of sports situations. In this study, we focused on the probability distribution regarding direction changes in chase-and-escape interactions and elucidated the corresponding spatiotemporal characteristics. For the spatial aspect, the frequency of the direction changes of the attacker (evader) was almost uniformly distributed. On the other hand, for the temporal aspect, the relative frequency of direction changes of the attacker showed a bimodal distribution.

The frequency of the direction changes of the attacker at each position on the court was approximately uniform. This spatial uniformity means that the probability of the evader changing or not changing its movement direction was almost constant regardless of its position on the court. This characteristic would be useful for the attacker to maximize the uncertainty in its direction change. The bias of the frequency of direction changes of the attacker rapidly approached a uniform distribution over time (see also Figs. S1, 2). This result indicates that the predictability for the defender using spatial probability information does not improve, even if information on the direction changes of the attacker had been accumulated. As a result, it would be difficult for the defender to anticipate in which direction the attacker would change its movement. Our results show that the proportion of direction change converging to a certain value is similar to matching pennies, in which players stochastically choose between two alternatives (heads or tails) with 50% in a mixed strategy Nash equilibrium in the framework of Game Theory^28–30^. These results suggest that the attacker randomizes its own actions so that they cannot be predicted by the defender.

The response time of the defender to the direction change of the attacker did not differ according to the position on the court and did not shorten over time (see also Figs. S3, 4). These results suggest that it would be difficult for the defender to respond predictively to the direction change of the attacker using situational probability information and support the idea that action selections with equal probabilities increase unpredictability against the opponent^28–30^.

The relative frequency of the time interval in the direction changes of the attacker showed a bimodal distribution. The peak of the distribution of short-interval direction changes was 200 ms, which, assuming that the human visuomotor delay is 200–300 ms, is not enough time for the attacker to gain feedback regarding the response of the defender to its own action^31^. Thus, this short-interval direction change (i.e., two consecutive directions) would be a feedforward control. In this case, the movement direction of the attacker in the *X* direction is the same as the original movement direction (i.e., right to left to right or vice versa), and thus, if the defender does not respond to the first direction change, the situation worsens for the attacker. Consequently, to execute two consecutive direction changes, the attacker relies on the defender responding to the first direction change. On the other hand, for long-interval direction changes, the attacker has enough time to gain feedback and can consider the observed information regarding the response of the defender. From a temporal standpoint, there are two types of attacker direction changes; namely, short-interval direction changes with feedforward control and long-interval direction changes with feedback control. In addition, the shapes of bimodal distribution were somewhat different between individuals but were approximately consistent within individuals, namely, between conditions (see Figs. S5, 6). This may reflect an individual playing preferences or habits.

The response of the defender to the short-interval direction change of the attacker was delayed compared with the response to the long-interval changes. Generally, it has been shown that humans respond quickly to frequent stimuli^32^. Based on this finding, the response time of the defender to short-interval direction changes should shorten, but contrarily, the response time lengthened in this study (see also Figs. S7, 8). One possible cause for this extra response delay is an interference of sensorimotor processing. In the double-stimulation paradigm, a phenomenon called the psychological refractory period has been demonstrated, in which the second response is delayed for two consecutive stimuli^20–24^. It is thought that this phenomenon is caused by overlapping of the response to the first stimulus and the response to the second stimulus. Because inter-stimulus intervals were distributed around 200 ms in the short-interval direction changes, a similar delay may occur to the response of the defender. In addition, it should be noted that, because the long-tailed distribution of long-interval changes increases the mean direction change time interval, this bimodal distribution can enhance the psychological refractory period effect by decreasing the temporal predictability of the direction changes. Interestingly, the temporal characteristics in bimodal distribution, which is a high frequency of direction change with short-interval and a long-tail of that with long intervals were more prominent in the group with high attack success rate than that with low attack success rate (see Fig. S9). These results indicate that the high frequency of direction change with a short interval is one of the characteristics of the high group, but the successful attack rate could not be explained by the frequency. This might be because it is more important to how combine the direction changes with short and long intervals rather than the frequency itself.

Our experiment was different from sport situations in several points. One was the viewpoint, that is, egocentric view in sport situations and bird’s eye view in our computer-based task. For example, in sport situations, when an attacker changes movement direction, the position of image would move from left to right (or vice versa) in the field of view of the defender. On the other hand, in our task, the position of the image in the field of view is little changed. That is, the body of defender acts as a reference frame in sport situations and does not act in our task. This difference in visual information could affect cognitive processing (e.g., S-R associations^33–37^) and could change the response time of defender. Another was the kinematic factors, that is, whole body movement in sport situations and finger movement in our task. In general, direction changes in locomotion are mechanically constrained^38–41^, and it cannot change movement direction suddenly in sport situations, unlike our task. This difference in mechanical constraints also could affect their interaction. Thus, further considerations by integrating sensory and motor factors would be necessary for a better understanding of effective attack behaviors in sport situations.

A limitation of the present study is the relatively small sample size. Regarding the result that the frequency of direction changes of the attacker at each position on the court was approximately uniform, it may be explained by the lack of samples. That is, we cannot exclude that absence of the significant effect of the *X* position and the interaction in the frequency of direction changes is associated with the number of participants in the present study. As a result, the result of the present study should be considered carefully, as it represents a pilot study.

In summary, our results showed that the decisions regarding direction changes of the evader have two characteristics: spatial uniformity and temporal bimodality. The former result is consistent with the findings of non-human studies that suggested the effectiveness of a strategy that increases unpredictability^42,43^, indicating that an element of unpredictability is key to successful escape across species. The latter result indicated that the attacker repeated effective behaviors (i.e., two consecutive direction changes), which lengthened the response time of the defender. Our results suggest that these characteristics could be useful for preventing the predictive response of the pursuer and to gain the benefit of an extra response delay of tens of milliseconds.

## Supplementary information

Supplementary Information

## Data Availability

The datasets generated and analyzed during the current study are available from the corresponding authors on reasonable request. The sample code for conducting the experimental task of the current study is available through 10.6084/m9.figshare.9784622.
