# Supplementary material for: Spatiotemporal characteristics of an attacker’s strategy to pass a defender effectively in a computer-based one-on-one task
Source: Sci Rep. 2019 Nov 21;9:17260. doi: 10.1038/s41598-019-54012-5 (PMC6872552; doi:10.1038/s41598-019-54012-5)
Supplement: Supplementary file 1 — Supplementary Information [file 41598_2019_54012_MOESM1_ESM.pdf]

Supplementary Information

Spatiotemporal characteristics of an attacker's strategy to pass a defender effectively in a computer-based one-on-one task

Kazushi Tsutsui, Masahiro Shinya, Kazutoshi Kudo

Corresponding authors:
Kazushi Tsutsui: k.tsutsui6@gmail.com
Kazutoshi Kudo: kudo@idaten.c.u-tokyo.ac.jp

This PDF file includes:
Figures S1 to S9

In Figures S1 to S8, participant 1 and 7, 2 and 8, 3 and 9. . . 6 and 12 were paired and competed each other. It should be note that, there were participants who started the trials from attacker or defender and the pairs that started from slow condition or fast condition in this study in order to counterbalance.

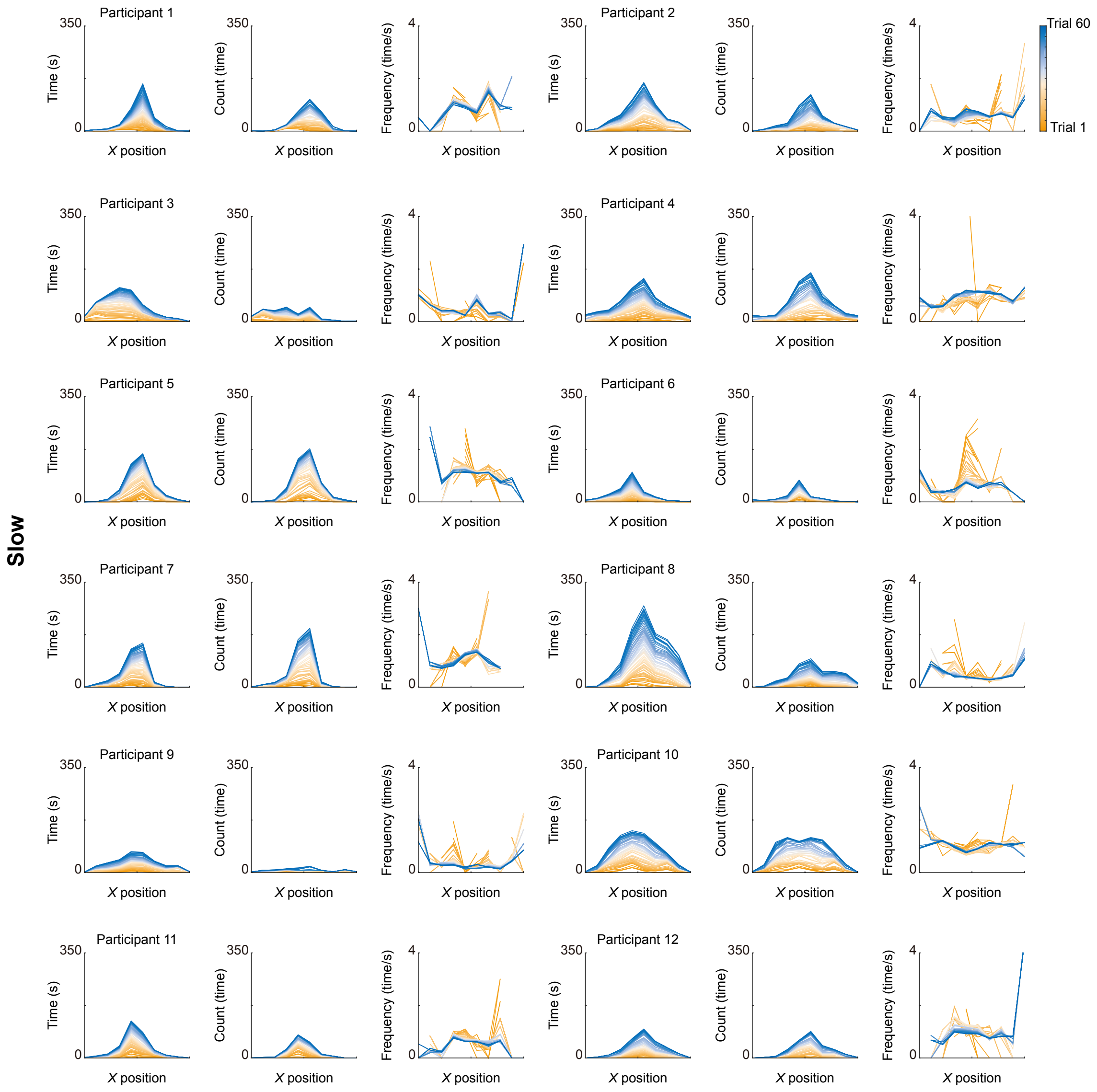

Figure S1. Cumulative changes in frequency regarding the direction changes of the attacker in each participant in slow condition.

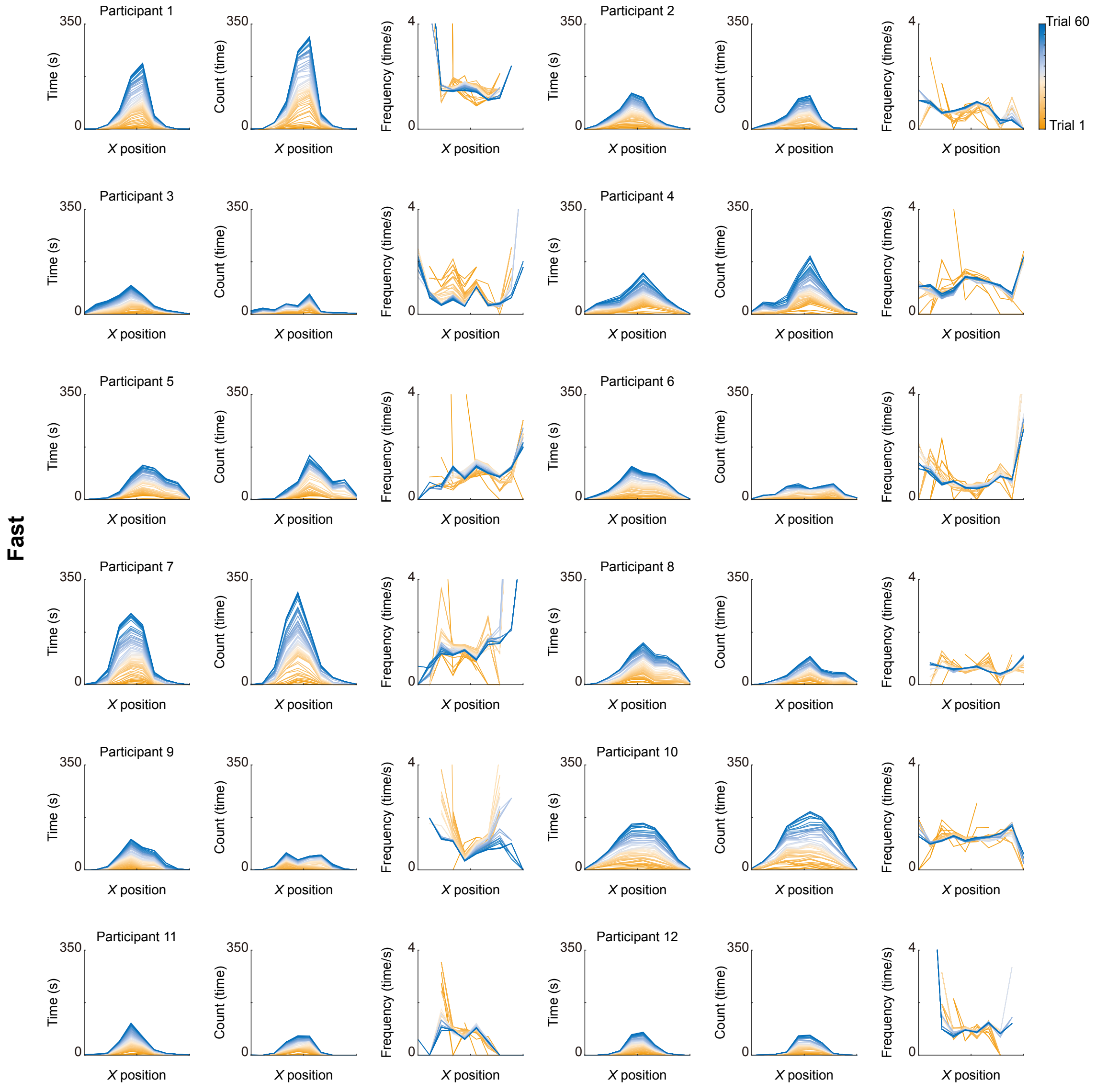

Figure S2. Cumulative changes in frequency regarding the direction changes of the attacker in each participant in fast condition.

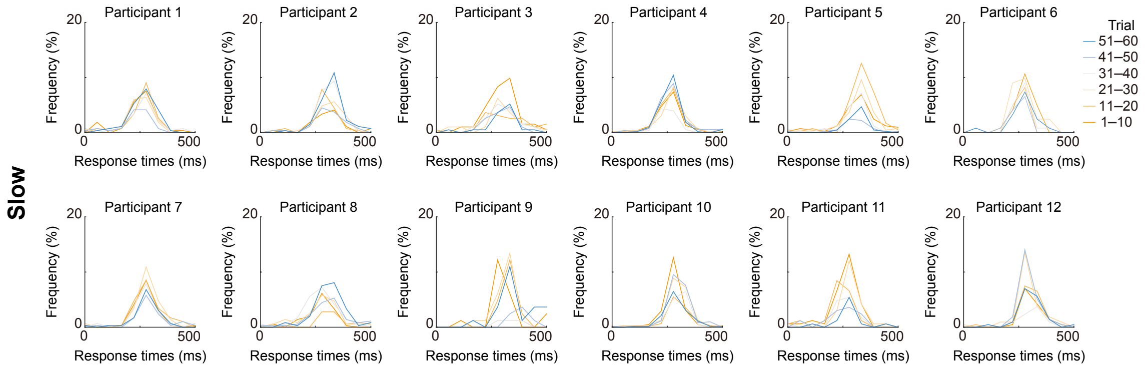

Figure S3. Histogram of the response time over time in each participant in slow condition. Each color represents a total of 10 trials.

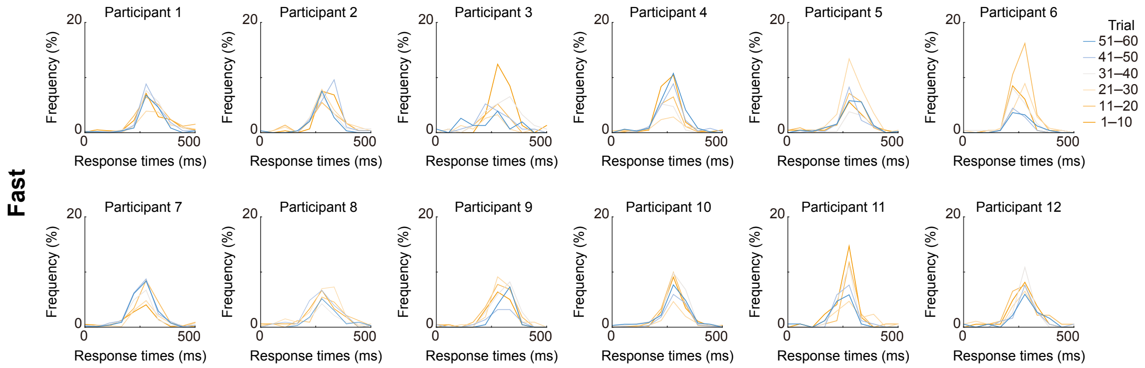

Figure S4. Histogram of the response time over time in each participant in fast condition.

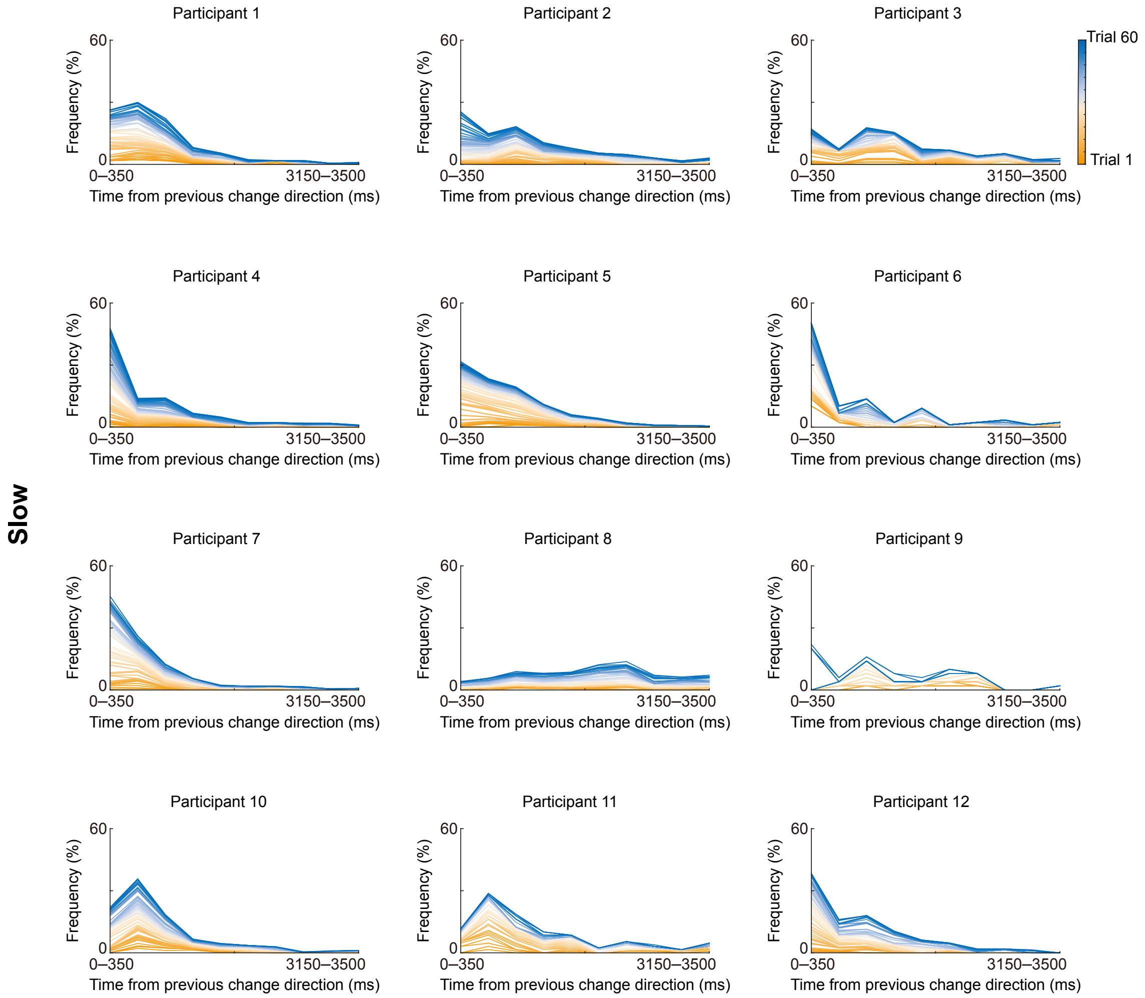

Figure S5. Cumulative changes of the time delay from the previous direction change in each participant in slow condition. The time range was set from 0 to 3500 ms, and the range was divided into 10 separate time bins.

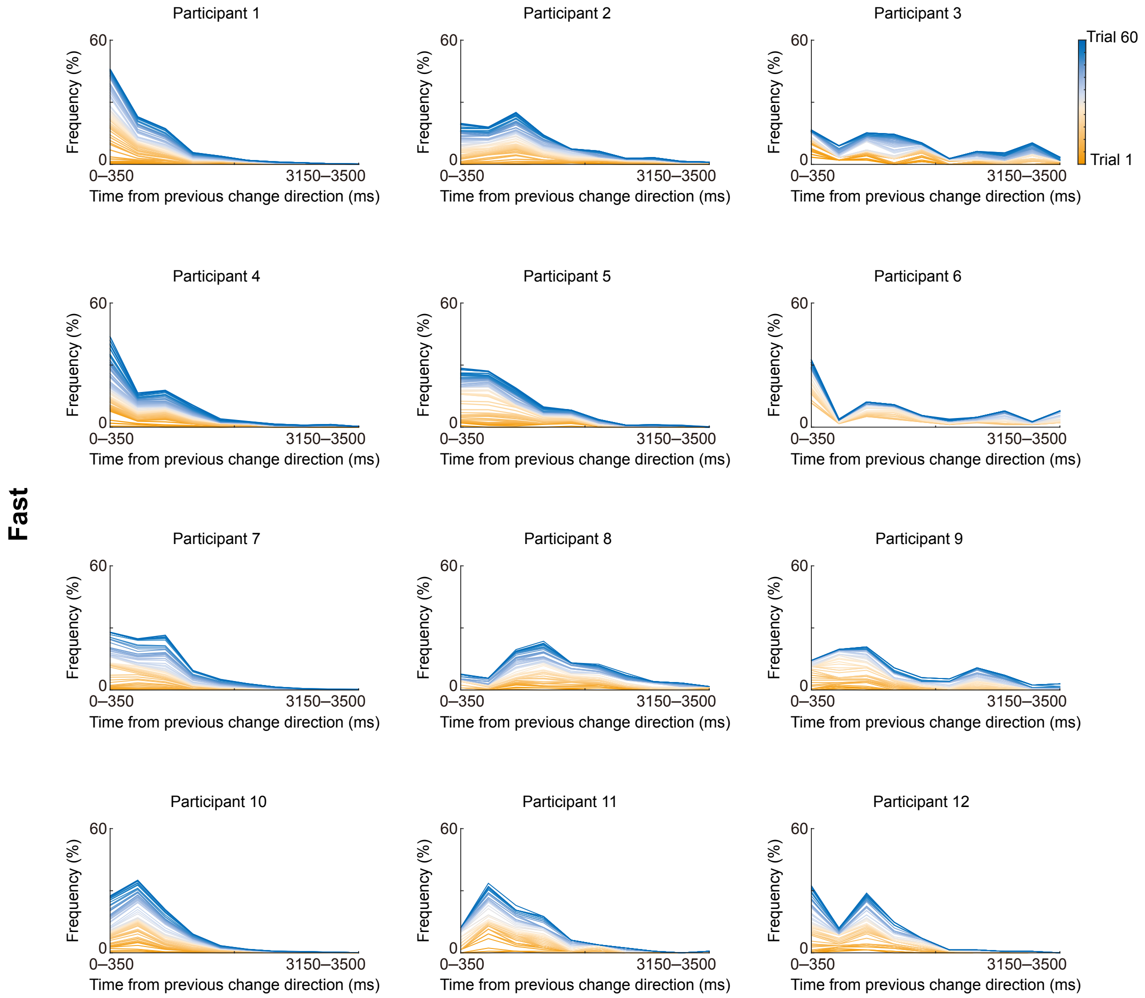

Figure S6. Cumulative changes of the time delay from the previous direction change in each participant in fast condition.

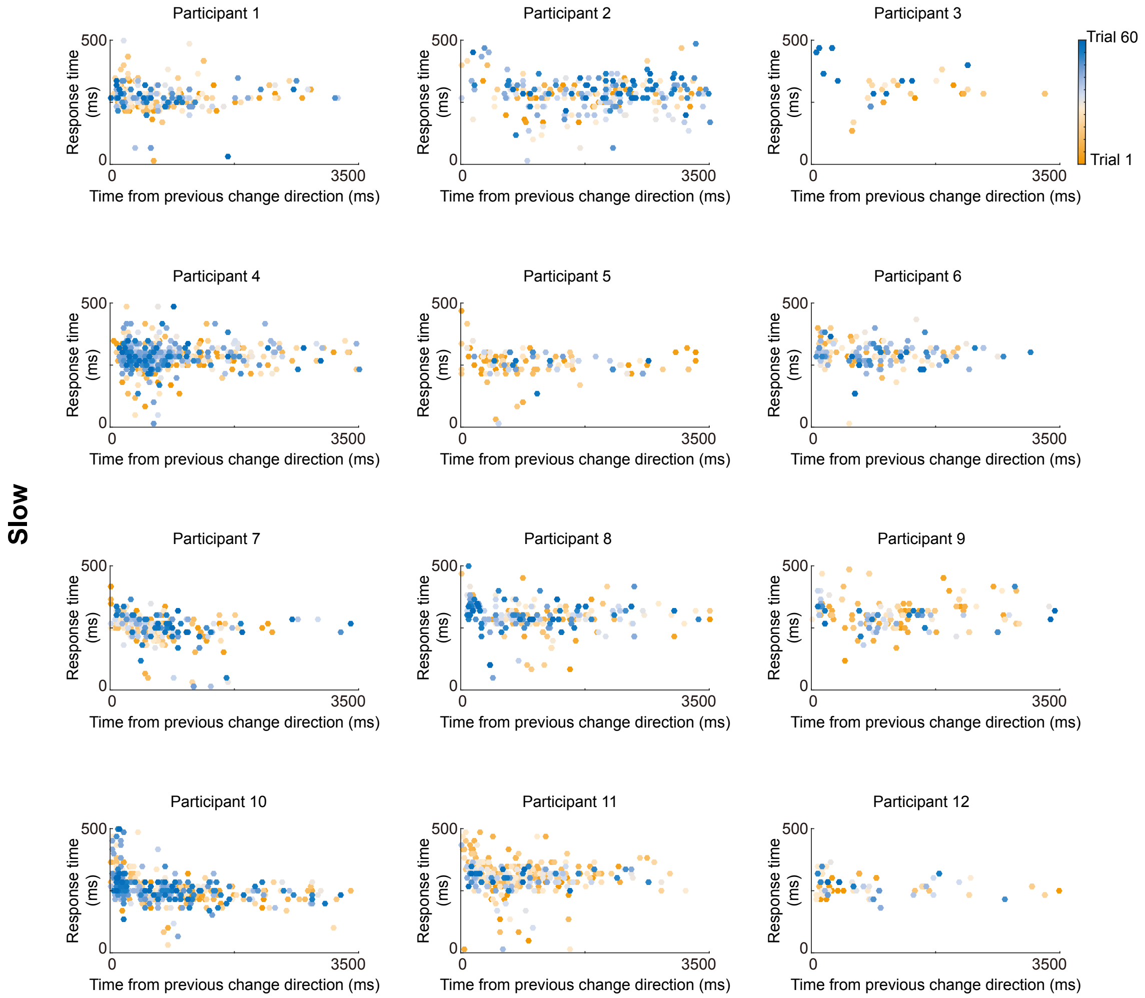

Figure S7.

The response time to direction change with each time interval in each participant in slow condition.

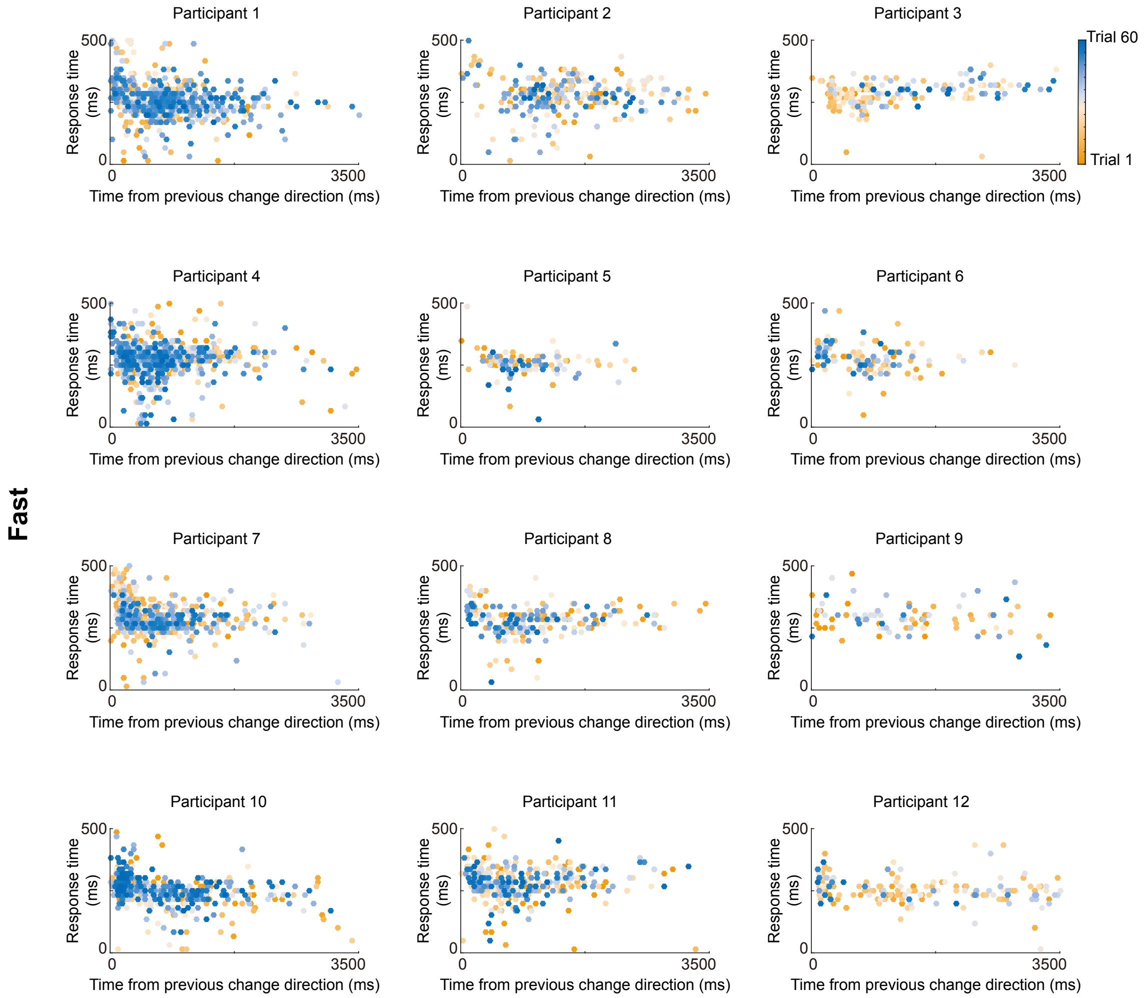

Figure S8. The response time to direction change with each time interval in each participant in fast condition.

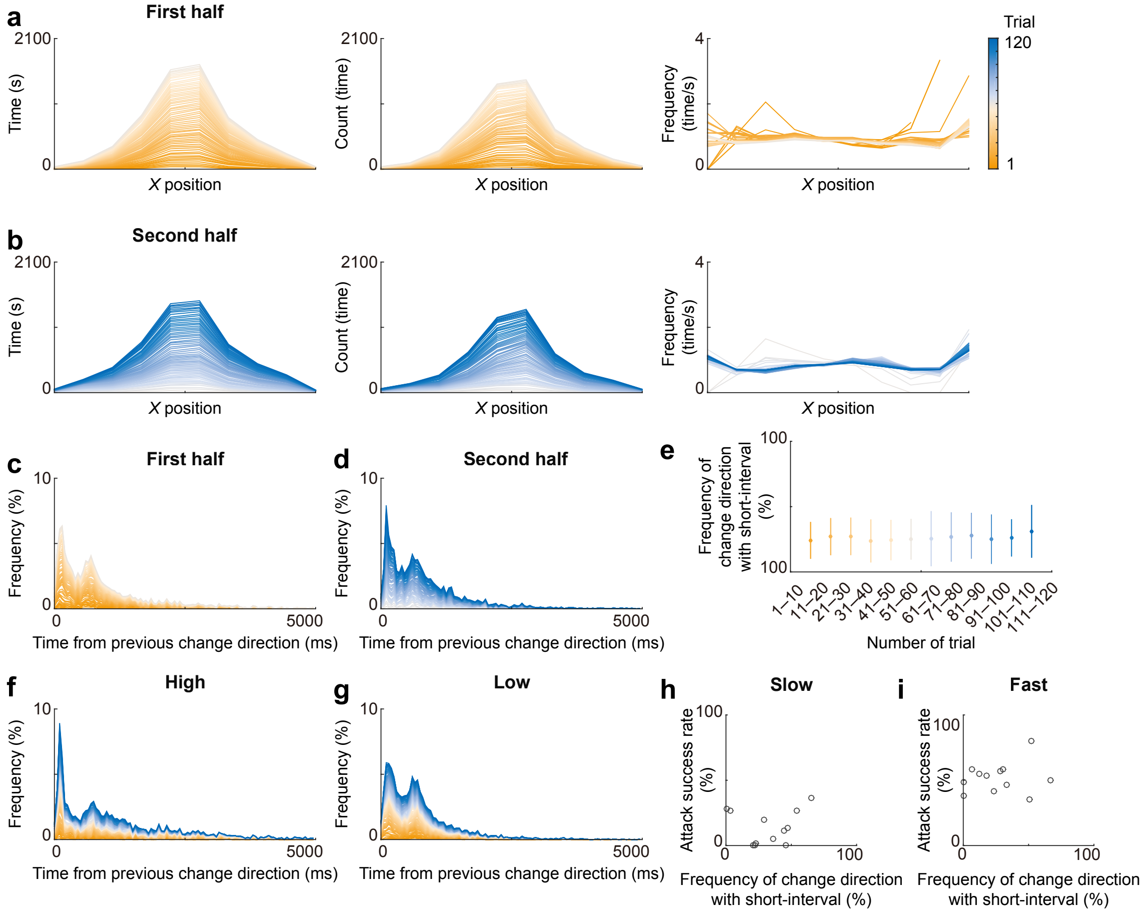

Figure S9. (a-d) The group data of cumulative changes in frequency regarding the direction changes in "First half (1 to 60 trials)" and "Second half (61 to 120 trials)" in each participant. (e) The group data of the frequency of change direction with short-interval in each 10 trials over time. There was no significant difference between time bins of ten trials ($F_{11,\,143} = 0.22$, $p = 0.99$, $\eta^2 = 0.018$). (f, g) The group data of cumulative changes of the time delay from the previous direction change in "High" and "Low" groups (each 6 participants) in successful attack rates using a median. (h, i) Correlations between the frequency of direction change with short-interval and successful attack rate. There were no statistically significant correlations ($r = 0.12$, $p = 0.72$ in slow condition; $r = 0.20$, $p = 0.54$ in fast condition; Figures c and d).
